# A randomized controlled trial comparing tacrolimus versus hydrocortisone for the treatment of atopic dermatitis in children: new perspectives on interferon gamma-induced protein and growth-related oncogene-α

**DOI:** 10.3389/fmed.2024.1399305

**Published:** 2024-07-24

**Authors:** Ammena Y. Binsaleh, Mostafa M. Bahaa, Thanaa A. Elmasry, Eman I. Elberri, Fedaa A. Kotkata, Eman El-Khateeb, Marwa Kamal, Marwa Ahmed El-samongy, Amir O. Hamouda, Amira M. Alghamdi, Sarah Alrubia, Muhammed M. Salahuddin, Nashwa Eltantawy

**Affiliations:** ^1^Department of Pharmacy Practice, College of Pharmacy, Princess Nourah Bint Abdulrahman University, Riyadh, Saudi Arabia; ^2^Pharmacy Practice Department, Faculty of Pharmacy, Horus University, New Damietta, Egypt; ^3^Pharmacology and Toxicology Department, Faculty of Pharmacy, Tanta University, Tanta, Egypt; ^4^Clinical Pharmacy Department, Faculty of Pharmacy, Tanta University, Tanta, Egypt; ^5^Department of Clinical Pharmacy, Faculty of Pharmacy, Fayoum University, Faiyum, Egypt; ^6^Department of Dermatology, Faculty of Medicine, Tanta University, Tanta, Egypt; ^7^Department of Biochemistry and Pharmacology, Faculty of Pharmacy, Horus University, New Damietta, Egypt; ^8^Department of Biochemistry, Faculty of Science, King Abdulaziz University, Jeddah, Saudi Arabia; ^9^Department of Pharmaceutical Chemistry, College of Pharmacy, King Saud University, Riyadh, Saudi Arabia; ^10^Department of Pharmacy Practice, Faculty of Pharmacy, Heliopolis University, Cairo, Egypt

**Keywords:** atopic dermatitis, hydrocortisone, tacrolimus, interferon gamma induced protein 10, GRO-α

## Abstract

**Introduction:**

Atopic dermatitis (AD) is a type of chronic inflammatory disorder that affects children.

**Aim:**

To investigate whether hydrocortisone or tacrolimus could be more effective for treating AD in children.

**Patients and methods:**

This clinical randomized investigation included 100 children with AD who met the eligibility criteria. AD patients were recruited from Tanta University’s Dermatology Department and divided into two groups (*n* = 50)., For four months, group 1 (the hydrocortisone group) received topical hydrocortisone cream. Group 2 received topical tacrolimus for four months. A dermatologist evaluated the patients at the start and four months after the treatment had been initiated to measure serum concentrations of neutrophil chemoattractant growth-related oncogene-α (GRO-α), interferon gamma induced protein 10 (IP-10), tumor necrosis factor alpha (TNF-α), vascular adhesion molecule 1 (VCAM-1), intercellular adhesion molecule 1 (ICAM-1). All patients were examined using the modified Eczema Area and Severity Index (mEASI) score.

**Results:**

Tacrolimus group showed a significant reduction in serum levels of all measured biomarkers (*p* < 0.05) when compared to its baseline and when compared to the hydrocortisone group. Both groups displayed a significant decline in mEASI score in comparison with their baseline values (*p* < 0.05).

**Conclusion:**

In children with AD, tacrolimus reduces inflammatory biomarkers better than hydrocortisone, suggesting its potential as a more effective treatment option.

**Clinical trial registration:**

https://clinicaltrials.gov, identifier NCT05607901.

## 1 Introduction

The most prevalent kind of chronic inflammatory skin disease is atopic dermatitis (AD) (1). About 80% of disease instances occur in infancy or childhood, with the remaining 20% occurring in adulthood. Between different countries, the disease prevalence for adults varies from 2.1 to 4.9%, while it varies from 2.7 to 20.1% for children (2). The natural course of the disease is highly variable, and individual outcomes are unexpected. Sensitive, dry skin, isolated or widespread eczematous lesions, and frequently extremely itchy skin are all symptoms of AD. The variable clinical phenotype is affected by age, severity, and ethnic origin (3).

Although the actual etiology of AD, a complex genetic disorder, is not fully understood, an interaction between inherited and environmental factors may contribute to its development (4). The two primary groups of genes involved are those that control the synthesis of cytokines required for an immunological response and those that code for structural proteins found in the epidermis and epithelial cells (5). Increasing T-helper 2 (TH2) activity results in the release of several interleukins such as interleukin (IL)-3, IL-13, IL-6, IL-10, and IL-5 in AD patients. It represents an imbalance in T-helper cell 1 (TH1) and TH2 immunological responses that can lead to blood eosinophilia, higher serum immunoglobulin (Ig) E levels, and accelerated mast cell development and growth (6).

The cornerstone of AD treatment against which other treatments are compared is topical corticosteroids (TCS), which reduce inflammation through different pathways (7). Even though TCS are extremely effective, they can also locally result in acne, rosacea, localized hypertrichosis, purpura, perioral dermatitis, telangiectasias, and striae. The hypothalamic-pituitary-adrenal axis can be suppressed by systemic absorption, along with infections, hyperglycemia, cataracts, glaucoma, and delays in development in children (8). Because these adverse effects are more likely to take place with ongoing use, it is crucial to explore alternate therapeutic options.

To overcome acute flares and minimize the severity of recurrent flares, two topical calcineurin inhibitors (TCI), tacrolimus and pimecrolimus, dampen the immune system and function as immunomodulators (9). They block the synthesis of pro-inflammatory cytokines such as IL-2, IL-3, IL-4, IL-17, and tumor necrosis factor alpha (TNF-α) by T cells and calcineurin, which inhibits T cell proliferation. According to previous reports, TCI showed more efficacy than TCS with fewer adverse effects (10–12). Therefore, TCI were regarded as a feasible substitute.

The tissue damage brought on by AD lesions may cause the release of neutrophil chemoattractant. Expression of neutrophil chemo-attractants has an impact on neutrophil activation, proliferation, and recruitment within AD lesions (13). A large proportion of CD4 T lymphocytes in AD patients contain chemokine receptor type 4 (CCR4) receptors, allowing them to bind to TH2-related chemokines such as thymus and activation-regulated chemokine (TARC) (13). Patients with AD have higher serum levels of TARC which are positively linked with the severity of the disease (14).

A chemokine associated with Th1 cells called Interferon-gamma (IFN- γ) induced protein 10 (IP-10) can be produced when IFN-γ is released by Th1 cells. As a result, IP-10 attracts and activates more stimulated lymphocytes (15). IP-10’s chemotactic activity contributes to both innate and adaptive immunity (15). Powerful neutrophil chemoattractant Growth-related oncogene-α (GRO-α) also plays a fundamental role in chronic inflammation and various autoimmune disorders (16).

The purpose of this study was to compare the efficacy and safety of topical tacrolimus ointment versus topical hydrocortisone cream in AD children. Tacrolimus and hydrocortisone’s ability to lower the inflammatory markers that are often high in AD patients has not been previously compared in investigations.

## 2 Patients and methods

From November 2022 to August 2023, the research was carried out at Tanta University’s Dermatology Department, Faculty of Medicine. This study involved 100 Outpatient Clinic patients who met the inclusion requirements. The study was approved by the Tanta University Faculty of Medicine’s National Research Ethics Committee under approval code 35928/10/2022. The Helsinki Declaration and its modifications from 1964 were followed in the study’s methodology and design. Patients were told that they might leave the trial at any time. “If they are able to appreciate the trial’s goals and dangers,” patients or their legal representatives have provided written informed consent.

### 2.1 Inclusion criteria

Male or female.

5–16 years old patients diagnosed according to Hanifin and Rajka criteria by dermatologist (17).

The capacity and desire to adhere to all study requirements, show up for all scheduled appointments and successfully finish the study.

### 2.2 Exclusion criteria

Patients using systemic or inhaled steroids.

Non-steroidal anti-inflammatory medication users.

Individuals receiving biological therapy or immunosuppressive medications for inflammatory bowel disease.

Patients who are taking any medications that could impact the serum levels of the monitored biomarkers.

Atopic dermatitis patients on systemic therapy within the past 4 weeks.

Women who are expecting a baby, nursing a baby, or who are planning a family but are not using a reliable method of birth control.

### 2.3 Study design

This study compared the safety and efficacy of tacrolimus with hydrocortisone in treating pediatric atopic dermatitis based on serum inflammatory biomarkers. It was a prospective, randomized, and double-blinded clinical trial.

This trial was registered as NCT05607901 at Clinical Trials.gov in 2022.

**FIGURE 1:**
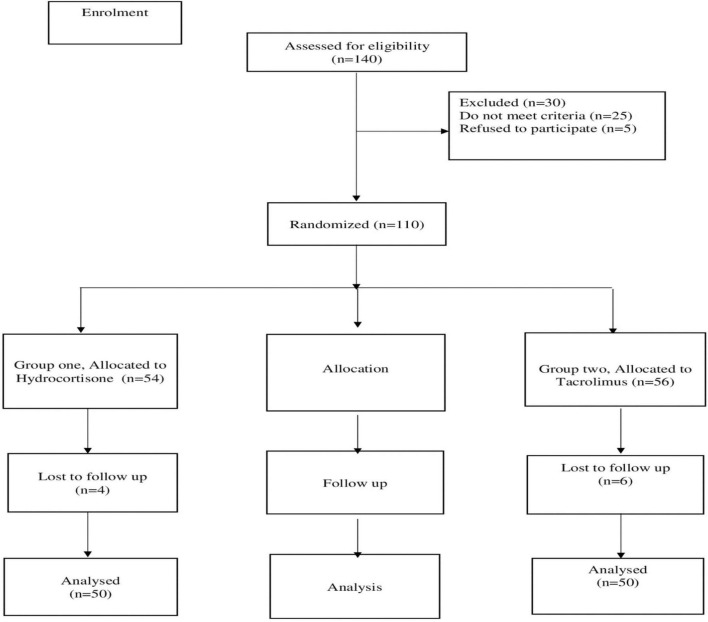
CONSORT diagram showing the flow of the patients during the study.

According to the CONSORT flow diagram in Figure 1, the participants were randomly divided into two groups (*n* = 50). The Recommended dose for tacrolimus and hydrocortisone cream was based on previous study (18). 4 months study duration was based on previous studies that investigated the use of TCS in AD (19, 20). A computer random number generator was used for selecting random permuted blocks for the randomization.

Group 1 (Hydrocortisone group): For four months, 50 patients will get treatment by applying a thin layer of 1% hydrocortisone cream twice daily to the affected areas (Multi-Apex, HydrocortR, Egypt).

Group 2 (Tacrolimus group): For four months, 50 patients will get treatment by applying a thin layer of 0.03% topical tacrolimus ointment twice daily to the affected areas (Tarolimus*^R^*, Andalous Pharma, Egypt).

Patients or their caregivers were taught to apply medication using the fingertip unit method (21) to avoid applying medication in too thin or too thick layer to avoid lack of response or increase risk of adverse effects, respectively. Participants, or their caregivers, were also instructed to avoid applying the cream to healthy skin or areas not affected by AD. Also, patients were advised to apply moisturizers during the study duration. Patients were advised to apply moisturizers (Just Vaseline) during the study which is applied within 3 min after bathing to keep skin hydration.

### 2.4 Biochemical analysis

Enzyme-Linked Immunosorbent Assay (ELISA) kits were used to analyze serum samples from each patient in line with the manufacturer’s instructions (Sunredio, Shanghai) for measuring serum intercellular adhesion molecule 1 (ICAM-1) (Kit Catalog No: 201-12-0213), serum vascular cell adhesion molecule 1 (VCAM-1) (Kit Catalog No: 201-12-0204), chemoattractant growth-related oncogene-α (GRO-α) (Kit Catalog No: 201-12-0061), interferon gamma induced protein 10 (IP-10) (Kit Catalog No: 201-12-4017), and TNF-α (Kit Catalog No: 201-12-0083).

### 2.5 Sample size calculation and blindness

Utilizing NCSS, LLC’s Power Analysis and Sample Size (PASS) Software, 15th edition (2017), Kaysville, Utah, USA, the sample size was determined.

Based on a previous study (22), A large effect size (Cohen’ dz = 0.8) was hypothesized for the biomarkers that will be used before and after treatments.

When the population effect size is 0.80 and the significance level (α) is 0.05, using a two-sided paired *t*-test, a sample size of 50 data pairs offers more than 80% power to reject the null hypothesis of zero effect size. To ensure proper treatment assignment, study medicines were given to participants by an unblinded pharmacist; the pharmacist was not involved in the evaluation of research outcomes. Both formulations were prepared to be identical in size and appearance of the containers (tubes) and labeling.

### 2.6 Clinical assessment

All patients underwent a dermatological examination using the modified Eczema Area and Severity Index (mEASI) score to assess the severity of dermatitis (23). Additionally, participants were routinely checked for the onset of any adverse outcomes during the duration of the study.

### 2.7 Outcomes

Primary outcome was the comparison of tacrolimus ointment versus topical hydrocortisone cream on the dermatitis severity scale and secondary outcome comparing the effect of the tacrolimus and hydrocortisone on serum biomarkers.

### 2.8 Statistical analysis

GraphPad Prism v9 (GraphPad software, Inc., San Diego, CA, USA), a statistical analysis programme, was used for the analyses. The normal distribution of continuous variables has been analyzed using the Shapiro–Wilk test. Significant differences within the group before and after therapy were determined using paired Student’s *t*-tests. To find significant variations between groups before and after therapy, unpaired Student’s *t*-tests were performed. In terms of numbers, qualitative variables were provided, while quantitative values were expressed as mean and SD. Using Pearson’s correlation coefficient, parameters were correlated. On categorical data, the Chi-square test and fisher exact test were applied. All *p*-values were two-tailed, with *p* < 0.05 considered statistically significant.

## 3 Results

### 3.1 Baseline demographic data

This study involved 100 AD patients who completed the study and assigned to one of two groups. For four months, group one received hydrocortisone cream; group two received tacrolimus ointment for four months. Six patients were lost to follow up in hydrocortisone group because they did not come to the university hospital in the second visit. Four patients were lost to follow up in tacrolimus group as two of them did not come to hospital in the second visit and two of them developed asthma that require inhaled steroids. Accordingly, 100 patients completed the study.

Table 1 displayed their baseline statistics. There were no significant differences in demographic data between the studied groups; age (*p* = 0.864), sex (*p* = 0.688), weight (*p* = 0.081), and height (*p* = 0.065).

**TABLE 1:** Clinical and demographic data in the two study groups.

Parameter	Hydrocortisone group	Tacrolimus group	*P*-value
Age (year)	11.75 (8–13.63)	12 (8–14)	0.864
Sex (M/F)	23/27	25/25	0.688
Height (m^2^)	1.425 (0.88–1.62)	1.6 (0.92–1.68)	0.065
Weight (kg)	47.50 (17.75- 60.25)	56.50 (20.75- 64.25)	0.081

Data are expressed as median, numbers, and interquartile range, M: Male, F: Female, Significance at (*p* < 0.05). Hydrocortisone group: 50 patients received hydrocortisone cream for four months, tacrolimus group: 50 patients received tacrolimus ointment for four months.

### 3.2 Effect of tacrolimus and hydrocortisone on serum biomarkers

Table 2 demonstrated no significant difference in baseline values between the two groups when comparing them using an unpaired *t*-test (*P* < 0.05).

**TABLE 2:** Analysis of inflammatory biomarkers in the two study groups.

Character	Hydrocortisone group			Tacrolimus group			^b^*p*-value
	Before treatment	After treatment	^a^*p*-value	Before treatment	After treatment	^a^*p*-value	After treatment
TNF-α (pg/ml)	171.6 ± 20.41	168.7 ± 18.44	0.304	173.4 ± 18.12	161.8 ± 17.35	< 0.0001	0.03
GRO-α (pg/ml)	87.06 ± 6.37	83.25 ± 7.44	0.003	88.85 ± 5.51	77.71 ± 7.072	< 0.0001	0.0002
IP-10 (pg/ml)	169.5 ± 20.87	164.4 ± 19.37	0.006	171.1 ± 21.54	156.6 ± 14.38	0.0001	0.02
ICAM-1 (pg/ml)	134.9 ± 14.56	132.5 ± 14.17	0.20	133.5 ± 16.86	126.7 ± 14.87	0.0001	0.04
VCAM-1 (ng/ml)	11.53 ± 2.253	10.87 ± 3.003	0.203	11.55 ± 2.66	9.762 ± 2.161	0.0002	0.03

Hydrocortisone group: 50 patients received hydrocortisone cream for four months, tacrolimus group: 50 patients received tacrolimus ointment for four months. Data are expressed as mean ± SD, TNF-α, tumor necrosis factor-alpha; GRO- α, neutrophil chemoattractant growth-related oncogene alpha; IP-10, interferon gamma induced protein; ICAM-1, intercellular adhesion molecule 1; VCAM, vascular cell adhesion molecule 1. ^a^significance within groups using paired *t*-test. ^b^significance between groups using unpaired *t*-test. Significance at (*p* < 0.05).

Regarding group 1, paired *t*-test showed that there were significant differences in all measured parameters when compared to baseline except for TNF-α, ICAM-1, and VCAM-1 as follows: GRO-α (*p* = 0.003), IP-10 (*p* = 0.006), ICAM-1 (*p* = 0.20), VCAM-1 (*p* = 0.203), and TNF-α (*p* = 0.304) (Table 2).

Regarding group 2, Table 2 revealed that all measured parameters had significant differences from their baseline values as follows: GRO-α (*p* ≤ 0.0001), IP-10 (*p* ≤ 0.0001), ICAM-1 (*p* ≤ 0.0001), VCAM-1 (*p* = 0.0002), and TNF-α (*p* ≤ 0.0001) using paired *t*-test.

Unpaired *t*-test showed that there were a statistically significant changes in all studied markers after four months of intervention, as follows: TNF-α (*p* = 0.03), GRO-α (*p* = 0.0002), IP-10 (*p* = 0.02), ICAM-1 (*p* = 0.04), and VCAM (*p* = 0.03) (Table 2).

### 3.3 Effect of studied medications on modified Eczema Area and Severity Index (mEASI) score

Baseline mEASI score, both groups were 31.64 ± 6.465 and 30.44 ± 8.409 (Mean ± SD) indicating that all patients were at severe grade according to Hanifin et al. (24).

Regarding hydrocortisone group, mEASI score was as follow, (31.64 ± 6.465 versus 11.64 ± 2.705, *p* = 0.000) using paired *t*-test.

Regarding tacrolimus group, mEASI score was as follow, (30.44 ± 8.409 versus 10.87 ± 2.535, *p* = 0.000) using paired *t*-test.

The surface area ranged from 10 to 40% of body surface area. Face, trunk and extremities were all included.

**FIGURE 2:**
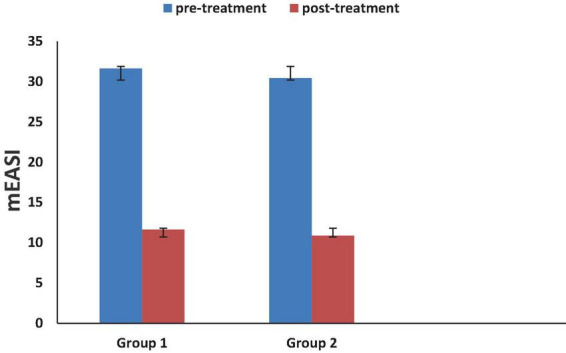
Effect of studied medications on modified Eczema Area and Severity Index (mEASI) score.

Figure 2 showed that both tacrolimus and hydrocortisone group significantly reduced mEASI score when compared to their baseline values. After treatment, hydrocortisone group responded earlier than tacrolimus group, but there was non-significant difference between the two study groups when compared to after treatment values using unpaired *t*-test (*p* = 0.145).

### 3.4 Correlation analysis

There was a significant correlation between mEASI and serum GRO-α (*r* = 0.445, *p* = 0.001), mEASI and IP-10 (*r* = 0.347, *p* = 0.007), and mEASI and VCAM (*r* = 0.368, *p* = 0.008).

### 3.5 Analysis of the drug related side effects

Table 3 showed that hydrocortisone produced significant skin atrophy (*p* = 0.021), and erythema (*p* = 0.045), hypopigmentation (*p* = 0.005) when compared to tacrolimus group. Tacrolimus group produced significant burning sensation (*p* = 0.01) when compared to hydrocortisone group.

**TABLE 3:** Comparison of drug-related adverse effects between the groups.

Side effect	Hydrocortisone group	Tacrolimus group	*P* value
Burning sensation	3	13	0.01
Skin atrophy	11	3	0.021
Erythema	14	5	0.045

Hydrocortisone group: 50 patients received hydrocortisone cream for four months, Tacrolimus group: 50 patients received tacrolimus ointment for four months. Data were presented as numbers. Significance at (*p* < 0.05).

## 4 Discussion

Children who have atopic dermatitis (AD) suffer from a persistent inflammatory condition. It is extremely itchy and frequently occurs in new-borns and kids, especially in people with atopy. Complex connections between susceptibility genes, the environment, and the pathophysiology of AD include epidermal barrier dysfunction, immunological responses to allergens, weakened antimicrobial defense, and immune responses to allergens (25).

To our knowledge, this was the first clinical study to compare between tacrolimus and hydrocortisone in AD and investigated their effect on serum GRO-α, and IP-10 in children. As AD is a progressive, painful, and itchy disease that requires immediate and effective management, this study did not include a placebo arm and hydrocortisone group was considered as a control. Although analysis of serum markers was not routinely used in patients with AD but it was obvious that these inflammatory markers were involved in the pathogenesis of AD and we analyzed these markers to evaluate biological efficacy of the studied medications. We conducted our study using the least potent steroid, hydrocortisone, to avoid the adverse effects of strong steroids such as decreasing adrenal gland cortisol production (26). Almost all cases are children and their skin barrier are not well developed, so systemic absorption may occur (27). Certainly, considering strong steroids for chronic use may lead to a positive therapeutic effect but may lead to severe side effects in these children and AD is relapsing in nature and application of strong steroid to wide area may lead to systemic absorption especially, AD affects flexures and systemic absorption is faster in this area (28).

Our results revealed that tacrolimus group showed statistically significant differences in serum IP-10, GRO- α, and TNF- α in comparison with its baseline and the hydrocortisone group. Our results came in accordance with previous studies (29–32). In lipopolysaccharide (LPS) activated TH-1 cells, tacrolimus decreased IP-10, GRO-α, and TNF-α expression in a time- and dose-dependent manner (29). Tacrolimus acts by preventing the activation of T lymphocytes and the production of TH2-related cytokines (IL-3, IL-4, IL-5, and IL-13) as well as Th1-related cytokines (GM-CSF, TGF-, IP-10, IL-12, IL-11, and IL-18) (33). Tacrolimus interacts with the FK506-binding protein, and the resulting complex prevents the activation of T cells and calcineurin phosphatase. Tacrolimus also inhibits T cells’ production of inflammatory cytokines such as TNF-α, IL-1, and IL-6 (34). Recent data indicates that chemokines may play a role in AD. The plasma of AD patients contains higher levels of TARC, a chemokine associated to Th2, and its level is strongly correlated with disease severity (35, 36). Activated lymphocytes are strongly chemotactically directed toward inflammatory sites, especially after infection, by the Th1-related chemokine IP-10 (37). Particularly following tissue damage, the chemoattractant GRO-α can cause neutrophil and T-lymphocyte chemotaxis (16). TNF-α, a cytokine that promotes inflammation, is crucial to the development of AD inflammation (38). In this work, we presented evidence that tacrolimus can inhibit cytokines and chemokines (GRO-α, IP-10) expression in AD children for the first time in the literature. These results imply that tacrolimus may treat AD by regulating the production of AD-related cytokines and chemokines in addition to suppressing T-cell activation. According to Sakuma et al. (39), tacrolimus had a suppressive effect on cytokine production that was more than that of alclometasone dipropionate and comparable to or greater than that of betamethasone valerate (30). In airway smooth muscle cells, corticosteroids inhibit growth-related oncogene protein-α through mitogen-activated kinase phosphatase-1 (40). Additionally, because IL-17 is a potent activator of GRO-α release, Laan et al. (39) observed that hydrocortisone inhibits IL-17-mediated IL-8 and GRO-α release. Sohn et al. (41) revealed the inhibitory effects of cortisone on IP-10 level in diabetic macular oedema, which is consistent with our results. Contrarily, topical steroids do not reduce the expression of the growth-related oncogene-α in nasal polyps, according to Cardell et al. (42). So, further studies are required to validate these results.

The tacrolimus group showed a significant reduction in serum ICAM-1 and VCAM-1 in comparison with its baseline and the hydrocortisone group. The hydrocortisone group did not show significant changes in these biomarkers. These findings are in line with other studies (43–46). The expression of VCAM-1 and ICAM-1 was significantly reduced in specimens that had been treated with tacrolimus (43). Contrarily, there was no discernible difference in the number and distribution of cells expressing adhesion molecules in biopsy samples taken from hydrocortisone-treated lesions (43). Adhesion molecules are recognized to play a significant role in allergic skin inflammation since they encourage the diapedesis of lymphocytes, monocytes, and granulocytes as well as the selective migration of memory T cells that express the cutaneous leucocyte antigen (CLA). In fact, endothelial leucocyte adhesion molecule-1 (ELAM-1), VCAM-1, and ICAM-1 and were found to be overexpressed in serum and tissue samples from AD patients (43). Tacrolimus may produce its effects by either decreasing the expression of adhesion molecules on the surface of endothelial cells or by decreasing the production of cytokines that are known to increase the expression of adhesion molecules, such as TNF-α, IF-γ, IL-4 and IL-13,2,3 (47, 48). Tacrolimus may lessen either the extravasation of T-lymphocytes, eosinophils, and neutrophils (via the downregulation of VCAM-1 and ICAM-1) or the endothelial rolling of CLA+ T lymphocytes (through the downregulation of ELAM-1) according to Caproni et al. (43). Caproni et al. (43) also reported that there are a number of reasons that could account for the hydrocortisone group’s inability to significantly reduce the adhesion molecules. Although tacrolimus and corticosteroids share the same route for inhibiting the nuclear factor of the activated T cell, their pharmacologic effects are likely due to additional genomic-independent processes. In example, tacrolimus has the potential to more powerfully and quickly control membrane receptors that can affect intracellular cascades (43). Additionally, there is ongoing debate regarding how glucocorticoids affect adhesion molecules. In spite of the fact that several research claimed glucocorticoids limit their expression (49, 50), others excluded this activity (51, 52).

Our research demonstrated that, after treatment, there was a statistically significant difference in mEASI when comparing tacrolimus and hydrocortisone groups to their baseline value but there was no statistical significance between the two study groups. These findings are in line with other studies (53, 54). In contrast, other studies reported a significantly greater decline in the tacrolimus group’s mEASI median percentage (18, 55, 56). The differences in outcomes between our research and the others might be explained by differences in patient age, demographic, and study period, and twice daily applications of hydrocortisone.

The current study revealed that hydrocortisone resulted in significant side effects more than tacrolimus. These results were matched and correlated with previous studies (54, 56). Others, reported that there tacrolimus group was higher than hydrocortisone group in terms of side effects (18). The application of twice daily hydrocortisone may be responsible for the higher incidence of side effects. Once daily application of hydrocortisone may lead to change in side effect profile between the two groups, but all cases in our study had severe grade of atopic dermatitis that require twice daily applications of least potent steroids such as hydrocortisone cream. It is well known that tacrolimus produces burning sensation when compared to hydrocortisone. Patients were advised to use it with gradual increase in the duration. In general, the burning sensation, resolve within one week of initiating topical tacrolimus and occur more frequently in adults than in children (57). These instructions may lead to decrease the incidence of burning sensation.

The current study revealed a significant positive correlation between mEASI, and GRO-α, IP-10, and VCAM. These findings are matched and correlated with previous studies (58–60).

## 5 Conclusion

We concluded from this randomized trial that tacrolimus 0.03% ointment is more beneficial than hydrocortisone cream in managing children with atopic dermatitis in terms of lowering the inflammatory markers, but there was no difference on the dermatitis severity scale. Moreover, tacrolimus has shown to be safer with a better side effect profile in comparison to hydrocortisone. To assess the adverse effect profile, further multicentre, long-term studies are needed.

## Data availability statement

The datasets presented in this article are not readily available because data is provided upon request due to privacy and ethical constraints. Requests to access the datasets should be directed to mbahaa@horus.edu.eg.

## Ethics statement

The studies involving humans were approved by the Tanta University Faculty of Medicine’s National Research Ethics Committee. The studies were conducted in accordance with the local legislation and institutional requirements. The participants provided their written informed consent to participate in this study.

## Author contributions

AB: Conceptualization, Data curation, Formal analysis, Funding acquisition, Investigation, Methodology, Project administration, Resources, Software, Supervision, Validation, Visualization, Writing – original draft, Writing – review & editing. MB: Conceptualization, Data curation, Formal analysis, Funding acquisition, Investigation, Methodology, Project administration, Resources, Software, Supervision, Validation, Visualization, Writing – original draft, Writing – review & editing. TE: Conceptualization, Data curation, Formal analysis, Funding acquisition, Investigation, Methodology, Project administration, Resources, Software, Supervision, Validation, Visualization, Writing – original draft, Writing – review & editing. EE: Conceptualization, Data curation, Formal analysis, Funding acquisition, Investigation, Methodology, Project administration, Resources, Software, Supervision, Validation, Visualization, Writing – original draft, Writing – review & editing. FK: Conceptualization, Data curation, Formal analysis, Funding acquisition, Investigation, Methodology, Project administration, Resources, Software, Supervision, Validation, Visualization, Writing – original draft, Writing – review & editing. EE-K: Conceptualization, Data curation, Formal analysis, Funding acquisition, Investigation, Methodology, Project administration, Resources, Software, Supervision, Validation, Visualization, Writing – original draft, Writing – review & editing. MK: Conceptualization, Data curation, Formal analysis, Funding acquisition, Investigation, Methodology, Project administration, Resources, Software, Supervision, Validation, Visualization, Writing – original draft, Writing – review & editing. ME-s: Conceptualization, Data curation, Formal analysis, Funding acquisition, Investigation, Methodology, Project administration, Resources, Software, Supervision, Validation, Visualization, Writing – original draft, Writing – review & editing. AH: Conceptualization, Data curation, Formal analysis, Funding acquisition, Investigation, Methodology, Project administration, Resources, Software, Supervision, Validation, Visualization, Writing – original draft, Writing – review & editing. AA: Conceptualization, Data curation, Formal analysis, Funding acquisition, Investigation, Methodology, Project administration, Resources, Software, Supervision, Validation, Visualization, Writing – original draft, Writing – review & editing. SA: Conceptualization, Data curation, Formal analysis, Funding acquisition, Investigation, Methodology, Project administration, Resources, Software, Supervision, Validation, Visualization, Writing – original draft, Writing – review & editing. MS: Conceptualization, Data curation, Formal analysis, Funding acquisition, Investigation, Methodology, Project administration, Resources, Software, Supervision, Validation, Visualization, Writing – original draft, Writing – review & editing. NE: Conceptualization, Data curation, Formal analysis, Funding acquisition, Investigation, Methodology, Project administration, Resources, Software, Supervision, Validation, Visualization, Writing – original draft, Writing – review & editing.
